# Covered Stent Disruption After Coronary Aneurysm Exclusion Revealed by Optical Coherence Tomography

**DOI:** 10.1016/j.jscai.2023.101129

**Published:** 2023-08-25

**Authors:** Franco Fabbiocchi, Giuseppe Calligaris, Antonio L. Bartorelli

**Affiliations:** a IRCCS Galeazzi-Sant'Ambrogio, Milan, Italy; b Centro Cardiologico Monzino, IRCCS, Milan, Italy; c Department of Biomedical and Clinical Sciences “Luigi Sacco,” University of Milan, Milan, Italy

**Keywords:** coronary aneurysm, covered stent, optical coherence tomography

**Figure 1:**
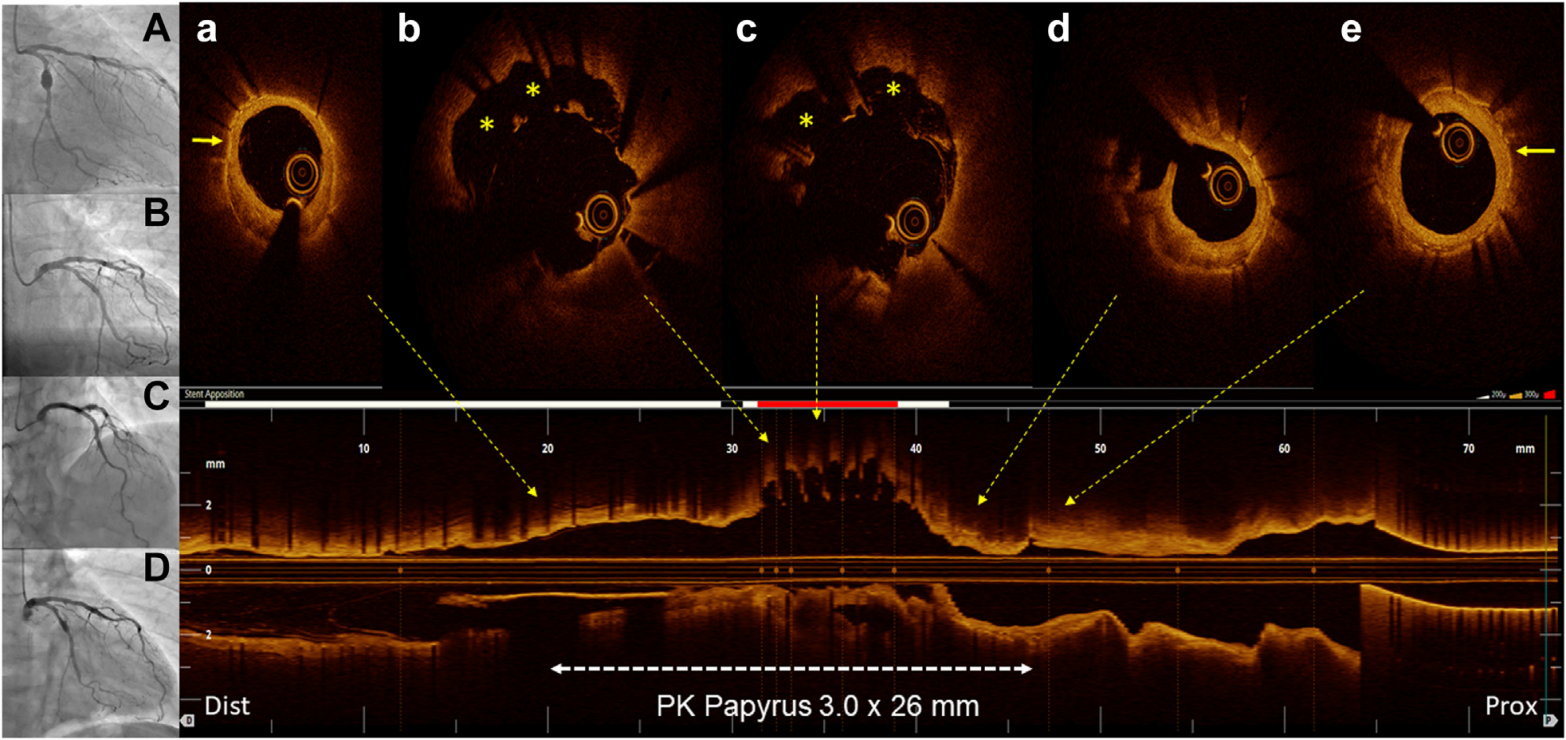
**Angiography evolution and optical coherence tomography pullback.** (**A**) Proximal (prox) left circumflex coronary artery coronary artery aneurysm (CAA). (**B**) CAA exclusion after Papyrus covered stent (CS) implantation in 2015. (**C, D**) Late CS middle segment malapposition in 2023. (**a, e**) CAA distal (dist) and prox sealing achieved by CS. Note that the membrane outside of the stent struts (arrow) is clearly visible. (**b, c**) CS membrane disruption and malapposition (asterisks). (**d**) In-stent restenosis.

A 62-year-old man, former smoker, hypertensive, and dyslipidemic, underwent coronary angiography in 2015 for exertional angina and positive stress test. In addition to a long stenosis of the mid left anterior descending artery, a saccular coronary artery aneurysm (CAA) of the proximal left circumflex coronary artery in between 2 stenoses was detected. After fixing the left anterior descending artery stenosis with a drug-eluting stent, CAA was treated with a 3.0 × 26 mm PK Papyrus polyurethane–covered stent (CS) (BIOTRONIK)^1^ distally connected to a drug-eluting stent. Postdilation at 16 atm with a 3.0 × 8 mm noncompliant balloon was performed. CAA was completely excluded, with no contrast surrounding the CS (Figure 1A, B). In 2023, patient recurred angina. Coronary angiography showed peristent contrast staining in the middle of the CS (Figure 1C, D). CS sealing of proximal and distal CAA ports, correct expansion, re-endothelialization, and proximal focal restenosis were detected by optical coherence tomography, along with a 180° arc of severe stent malapposition in the middle portion. Stent struts not bounded by the polyurethane membrane nor covered by neointima on the luminal side were visible, revealing disruption of long sections of the outer membrane otherwise clearly detectable where the struts were apposed (Figure 1A, E). We treated the restenosis with a drug-coated balloon, but we did not touch the malapposition concerned about the implant of another CS inside the first one.

This is the first report of CS failure due to spontaneous disruption of the elastic membrane. Although a manufacturing defect cannot be ruled out, the intrinsic features of the Papyrus, consisting of a polyurethane membrane sewn on the outside of a single metallic platform, could have played a role. Optical coherence tomography imaging had paramount value in revealing failure mechanisms, ruling out the possibility of a noncoverage of the proximal and distal ports of the aneurysm. The use of intravascular imaging may, therefore, be essential when placing a CS to maximize the chance of “CAA sealing.”
